# ﻿Molecular phylogeny and morphology reveal four new wood-inhabiting fungi of *Asterostroma* and *Radulomyces* (Basidiomycota) from Southwestern China

**DOI:** 10.3897/mycokeys.112.137098

**Published:** 2025-01-08

**Authors:** Junhong Dong, Qiaohua Deng, Minglan Chen, Daxiang Chen, Chunqin Zhou, Changlin Zhao

**Affiliations:** 1 College of Forestry, Southwest Forestry University, Kunming 650224, China Southwest Forestry University Kunming China; 2 Tongbiguan Provincial Nature Reserve, Mangshi 678499, China Tongbiguan Provincial Nature Reserve Mangshi China; 3 Management and Conservation Bureau, Yunnan Wumeng Mountain National Nature Reserve, Zhaotong, 657000, China Management and Conservation Bureau, Yunnan Wumeng Mountain National Nature Reserve Zhaotong China; 4 Yunnan Key Laboratory of Gastrodia and Fungal Symbiotic Biology, Zhaotong University, Zhaotong 657000, China Zhaotong University Zhaotong China

**Keywords:** Biodiversity, molecular systematics, new species, taxonomy, wood-decaying fungi, Yunnan Province

## Abstract

In the ecosystem, wood-inhabiting fungi play an indispensable role in wood degradation and the cycle of substances. They are regarded as the “key player” in the process of wood decomposition because of their ability to produce various enzymes that break down woody lignin, cellulose, and hemicellulose. In this study, four new wood-inhabiting fungal species, *Asterostromaparamuscicola*, *Radulomycesbambusinus*, *R.fissuratus*, and *R.sinensis*, were collected from southwestern China and were proposed based on the morphological and molecular evidence. *Asterostromaparamuscicola* is characterised by the felted-membranous to pellicular basidiomata with pinkish to slightly salmon-buff, a smooth hymenial surface, a monomitic hyphal system, and generative hyphae bearing simple-septate and subglobose, thin-walled, echinulate basidiospores measuring as 8–8.8 × 7–8 µm. *Radulomycesbambusinus* is characterised by the resupinate basidiomata with pinkish-white to pink, a tuberculate hymenial surface, a monomitic hyphal system and generative hyphae bearing clamp connections, and subglobose, slightly thick-walled, smooth basidiospores measuring as 6–7.5 × 5.5–7.3 µm. *Radulomycesfissuratus* is characterised by the coriaceous basidiomata with grey to grey-buff, a tuberculate hymenial surface, a monomitic hyphal system and generative hyphae bearing clamp connections, and globose, slightly thick-walled, smooth basidiospores measuring as 7–9.5 × 6.5–8.5 µm. *Radulomycessinensis* is characterised by the coriaceous basidiomata with straw to cinnamon to ocherous, a tuberculate hymenial surface, a monomitic hyphal system and generative hyphae bearing clamp connections, and broadly ellipsoid, slightly thick-walled, smooth basidiospores measuring as 7.5–9 × 6.2–7.5 µm. Sequences of the internal transcribed spacer (ITS) and large subunit (nrLSU) markers of the studied samples were generated, and phylogenetic analyses were performed with maximum likelihood, maximum parsimony, and Bayesian inference methods. Phylogenetic analyses of ITS+nrLSU nuclear RNA gene regions showed that four new species were assigned to the genera *Asterostroma* and *Radulomyces*. The phylogenetic tree inferred from the ITS sequences revealed that *A.paramuscicola* was closely associated with *A.macrosporum* and *A.muscicola*. Based on the ITS sequences, the topology showed that *Radulomycesbambusinus* was retrieved as a sister to *R.zixishanensis*. The taxon *R.fissuratus* forms a monophyletic lineage. The other one species, *R.sinensis*, was closely associated with *R.molaris* and *R.yunnanensis*.

## ﻿Introduction

The term “eukaryote” refers to cell structure and means that an organism’s genetic information is housed inside a structure called a nucleus (Money 2016). Fungi are eukaryotic microorganisms that play key ecological roles as decomposers, mutualists, or pathogens (Hyde et al. 2021). Fungi are a distinct, diverse, and ecologically important branch of the tree of life (James et al. 2020). Classification of the fungi has proven challenging due to the small number of known as compared to estimated species and a lack of sequence data for many extant taxa (Hyde et al. 2021). The phylum Basidiomycota constitutes a major group of the kingdom fungi and is second in species numbers to the phylum Ascomycota (Wijayawardene et al. 2018). Wood-inhabiting fungi have important industrial, medicinal, edible, and economic values, and a small number of them contain toxic metabolites (Niego et al. 2023).

The genus *Asterostroma* Massee, belonging to the family Peniophoraceae (Russulales, Basidiomycota), is typified by *A.apalum* (Berk. & Broome) Massee, and it is characterised by the resupinate, effused, brittle, loosely adnate, membranaceous to pellic basidiomata; a cream to ochraceous, smooth hymenial surface; a dimitic hyphal system with simple-septate on generative hyphae and dextrinoid asterosetae; present gloeocystidia; utriform basidia with 4-sterigmata and a basal simple septum; and subglobose to ellipsoid, smooth or tuberculate, amyloid basidiospores (Massee 1889; Bernicchia and Gorjón 2010). Based on the MycoBank database (http://www.mycobank.org, accessed on 9 November 2024) and the Index Fungorum (http://www.indexfungorum.org, accessed on 9 November 2024), the genus *Asterostroma* has registered 41 specific and infraspecific names, and six have been recorded from China (Liu et al. 2017; Deng et al. 2024; Dong et al. 2024a; Zhou et al. 2024).

The genus *Radulomyces* M.P. Christ., belonging to the family Radulomycetaceae (Agaricales, Basidiomycota), is typified by *R.confluens* (Fr.) M.P. Christ., and it is characterised by the resupinate, adnate, effused, ceraceous, hygrophanous basidiomata, smooth, tuberculate, odontioid to raduloid hymenial surface, a monomitic hyphal system with clamp connections on generative hyphae, clavate, sinuous basidia with 4-sterigmata and a basal clamp connection, with abundant oil drops, and ellipsoid to globose, smooth or minutely ornamented (spore dimorphism occurs in some species), slightly thick-walled, acyanophilous basidiospores (Christiansen 1960; Bernicchia and Gorjón 2010). Based on the MycoBank database (http://www.mycobank.org, accessed on 9 November 2024) and the Index Fungorum (http://www.indexfungorum.org, accessed on 9 November 2024), the genus *Radulomyces* has registered 30 specific and infraspecific names, and three have been found from China (Dong et al. 2024b).

Based on the morphological characteristics of asterosetae, the genus *Asterostroma* was placed in the family Lachnocladiaceae D.A. Reid (Parmasto 1971; Hallenberg and Eriksson 1985). Later, *Asterostroma* belonged to the russuloid lineage and was located in the family Peniophoraceae Lotsy in their phylogenetic analysis of ITS+nrLSU (Larsson and Larsson 2003; Miller et al. 2006; Larsson 2007; Liu et al. 2017). According to the morphological characteristics of the basidiospores in *Asterostroma* (Parmasto 1971; Boidin et al. 1997), this genus was divided into two subgenera: *Austroasterostroma* Parmasto (smooth and amyloid basidiospores) and *Asterostroma* ornamented and amyloid basidiospores (Liu et al. 2017; Zhou et al. 2024). Recently, phylogenetic analyses on *Asterostroma* from China based on ITS+nrLSU sequences and morphology have identified three new species: *A.rhizomorpharum* H.M. Zhou & C.L. Zhao, *A.roseoalbum* J.H. Dong & C.L. Zhao and *A.yunnanense* Y.L. Deng & C.L. Zhao were described (Deng et al. 2024; Dong et al. 2024a; Zhou et al. 2024).

The placement of two genera, *Aphanobasidium* Jülich and *Radulomyces*, was previously located in the family Pterulaceae Corner by the phylogenetic reconstructions of corticioid taxa (Larsson et al. 2004; Larsson 2007). Phylogenetically, *Radulomyces* belonged in the Pterulaceae (Agaricales) and was most closely related to *Radulotubus* Y.C. Dai, S.H. He & C.L. Zhao and *Aphanobasidium* (Zhao et al. 2016). These three genera form a strongly supported clade sister to the *Pterula-Deflexula-Pterulicium-Merulicium-Coronicium* clade based on sequence analyses of ITS+nrLSU (Zhao et al. 2016; Wang et al. 2018). Radulomycetaceae was characterized by the combination of resupinate basidiomes, a monomitic hyphal system, and the absence of cystidia, in which the phylogenetic analyses strongly support the segregation of Radulomycetaceae from Pterulaceae (Leal-Dutra et al. 2020). Based on the phylogenetic and morphological analysis, Leal-Dutra et al. (2020) revealed that no members of the three genera (i.e., *Aphanobasidium*, *Radulomyces*, and *Radulotubus*) within this superclade were pteruloid (i.e., coralloid basidiomes with a dimitic hyphal system) in their morphology. They were distinct from nearly all the other members of Pterulaceae, clearly by morphology and phylogeny, and then consequently Leal-Dutra et al. (2020) proposed the new family Radulomycetaceae to accommodate the three genera. Based on the ITS+nrLSU sequence and morphological characteristics, three new species, *R.hydnoides* J.H. Dong & C.L. Zhao, *R.yunnanensis* J.H. Dong & C.L. Zhao, and *R.zixishanensis* J.H. Dong & C.L. Zhao, were introduced from China (Dong et al. 2024b).

During investigations on wood-inhabiting fungi in southwestern China, some specimens were collected. To clarify the placement and relationships of these specimens, we carried out a phylogenetic and taxonomic study on the genera *Asterostroma* and *Radulomyces* based on the ITS and ITS+nrLSU sequences. These specimens were assigned to the genera *Asterostroma* and *Radulomyces*. Therefore, four new species, *Asterostromaparamuscicola*, *R.bambusinus*, *R.fissuratus*, and *R.sinensis*, are proposed, based on the morphological characteristics and phylogenetic analyses.

## ﻿Materials and methods

### ﻿Sample collection and herbarium specimen preparation

The fresh fruiting bodies on the dead bamboo and fallen angiosperm branches were collected from Dehong, Lincang, Pu’er, and Zhaotong of Yunnan Province, China. The samples were photographed in situ, and fresh macroscopic details were recorded. Photographs were recorded by a Nikon D7100 camera. All the photos were focus-stacked using Helicon Focus software. Macroscopic details and collection information (Rathnayaka et al. 2024) were taken and transported to a field station where the fruit body was dried on an electronic food dryer at 45 °C (Hu et al. 2022). Once dried, the specimens were sealed in an envelope and zip-lock plastic bags and labeled. The dried specimens were deposited in the herbarium of the Southwest Forestry University (SWFC), Kunming, Yunnan Province, China.

### ﻿Morphology

The macromorphological descriptions were based on field notes and photos captured in the field and lab. The color terminology follows Petersen (1996). The micromorphological data were obtained from the dried specimens after observation under a light microscope with a magnification of 10 × 100 oil. Sections were mounted in 5% KOH and 2% phloxine B (C_20_H_2_Br_4_Cl_4_Na_2_O_5_), and other reagents were also used, including cotton blue and Melzer’s reagent to observe micromorphology following Wu et al. (2022). To show the variation in spore sizes, 5% of measurements were excluded from each end of the range and shown in parentheses. At least thirty basidiospores from each specimen were measured. Stalks were excluded from basidia measurements, and the hilar appendage was excluded from basidiospore measurements. The following abbreviations are used: KOH = 5% potassium hydroxide water solution, CB– = acyanophilous, IKI– = both inamyloid and non-dextrinoid, L = mean spore length (arithmetic average for all spores), W = mean spore width (arithmetic average for all spores), Q = variation in the L/W ratios between the specimens studied, Q_m_ represented the average Q of basidiospores measured ± standard deviation, and n = a/b (number of spores (a) measured from given number (b) of specimens).

### ﻿Molecular phylogeny

The CTAB rapid plant genome extraction kit-DN14 (Aidlab Biotechnologies Co., Ltd., Beijing, China) was used to extract genomic DNA from the dried specimens according to the manufacturer’s instructions. The nuclear ribosomal internal transcribed spacer (ITS) region was amplified with ITS5 and ITS4 primers (White et al. 1990). The nuclear large subunit (nrLSU) region was amplified with the LR0R and LR7 primer pair (Vilgalys and Hester 1990; Rehner and Samuels 1994). The PCR procedure for ITS was as follows: initial denaturation at 95 °C for 3 min, followed by 35 cycles at 94 °C for 40 s, 58 °C for 45 s, and 72 °C for 1 min, and a final extension of 72 °C for 10 min. The PCR procedure for nrLSU was as follows: initial denaturation at 94 °C for 1 min, followed by 35 cycles at 94 °C for 30 s, 48 °C for 1 min, and 72 °C for 1.5 min, and a final extension of 72 °C for 10 min. The PCR products were purified and sequenced at Kunming Tsingke Biological Technology Limited Company (Yunnan Province, P.R. China). The newly generated sequences were deposited in NCBI GenBank (Table 1).

**Table 1.** List of species, specimens, and GenBank accession numbers of sequences used in this study. [New species is shown in bold; * type material].

Species Name	Sample No.	GenBank Accession No.		Country	References
		ITS	nrLSU		
*Aphanobasidiumpseudotsugae*	HHB-822	GU187509	GU187567	USA	Larsson (2007)
*Aphanobasidiumpseudotsugae*	UC 2023153	KP814353	AY586696	Sweden	Larsson (2007)
*Asterostromaandinum*	He 20120921–17	—	KY263874	China	Liu et al. (2017)
*Asterostromaandinum*	HHB-8546-sp	—	AF518600	USA	Hibbett and Binder (2002)
*Asterostromabambusicola*	He 4128	KY263864	—	Thailand	Liu et al. (2017)
*Asterostromabambusicola*	He 4132	KY263865	KY263871	Thailand	Liu et al. (2017)
*Asterostromacervicolor*	He 2314	KY263860	KY263868	China	Liu et al. (2017)
*Asterostromacervicolor*	He 4020	KY263859	KY263869	China	Liu et al. (2017)
*Asterostromacervicolor*	KHL 9239	AF506408	AF506408	Puerto Rico	Larsson and Larsson (2003)
*Asterostromacervicolor*	TMI 21362	AB439560	—	Japan	Larsson and Larsson (2003)
*Asterostromalaxum*	EL 33-99	AF506410	AF506410	Estonia	Larsson and Larsson (2003)
*Asterostromamacrosporum*	TMI 25696	AB439544	—	Japan	Suhara et al. (2010b)
*Asterostromamacrosporum*	TMI 25697	AB439545	—	Japan	Suhara et al. (2010b)
*Asterostromamedium*	HFRG_EJ220212_2_FRDBI 23891920	OQ133615	—	UK	Deng et al. (2024)
*Asterostromamedium*	HFRG_EJ210127_2FRDBI 18772203	OL828779	—	UK	Deng et al. (2024)
*Asterostromamuscicola*	He 4106	KY263861	KY263873	Thailand	Liu et al. (2017)
*Asterostromamuscicola*	He 20121104-1	KY263862	KY263872	China	Liu et al. (2017)
*Asterostromamuscicola*	TUMH 10017	AB439552	—	Japan	Suhara et al. (2010b)
*Asterostromaochroleucum*	HB 9/89	—	AF323737	Germany	Wagner (2001)
***Asterostromaparamuscicola***	**CLZhao 8594**	**PP392895**	**PQ306584**	**China**	**Present study**
*Asterostromarhizomorpharum*	CLZhao 31212	OR672732	OR879302	China	Zhou et al. (2024)
*Asterostromarhizomorpharum*	CLZhao 31216	OR672733	—	China	Zhou et al. (2024)
*Asterostromavararioides*	He 4136	KY263866	—	Thailand	Liu et al. (2017)
*Asterostromavararioides*	He 4140	KY263867	KY263870	Thailand	Liu et al. (2017)
*Asterostromayunnanense*	CLZhao 22781	OR048809	OR506285	China	Deng et al. (2024)
*Asterostromayunnanense*	CLZhao 22786	OR048811	OR506286	China	Deng et al. (2024)
*Confertobasidiumolivaceoalbum*	FP 90196	AF511648	AF511648	USA	Larsson and Larsson (2003)
*Dichostereumdurum*	FG 1985	AF506429	AF506429	France	Larsson and Larsson (2003)
*Dichostereumeffuscatum*	GG 930915	AF506390	AF506390	France	Larsson and Larsson (2003)
*Gloiothelelactescens*	EL 8-98	AF506453	AF506453	Sweden	Larsson and Larsson (2003)
*Gloiothelelamellosa*	KHL 11031	AF506454	AF506454	USA	Larsson and Larsson (2003)
*Meruliciumfusisporum*	Hjm s.n.	EU118647	EU118647	Sweden	Larsson (2007)
*Peniophoracinerea*	NH 9808/1788	AF506424	AF506424	Spain	Larsson and Larsson (2003)
*Peniophoraincarnata*	NH 10271/1909	AF506425	AF506425	Denmark	Larsson and Larsson (2003)
*Pterulaecho*	AFTOL-ID711	DQ494693	AY629315	USA	Larsson and Larsson (2003)
***Radulomycesbambusinus***	**CLZhao 35383** *	**PQ306582**	**PQ306589**	**China**	**Present study**
***Radulomycesbambusinus***	**CLZhao 35384**	**PQ306583**	**PQ306590**	**China**	**Present study**
*Radulomycesconfluens*	Cui 5977	KU535661	KU535669	China	Wang et al. (2018)
*Radulomycesconfluens*	He 2224	KU535662	KU535670	China	Wang et al. (2018)
*Radulomycescopelandii*	Dai 15061	KU535664	KU535672	China	Wang et al. (2018)
*Radulomycescopelandii*	Wu 9606-5	KU535663	KU535671	China	Wang et al. (2018)
***Radulomycesfissuratus***	**CLZhao 29670** *	**PQ306579**	**PQ306586**	**China**	**Present study**
***Radulomycesfissuratus***	**CLZhao 29695**	**PQ306580**	**PQ306587**	**China**	**Present study**
***Radulomycesfissuratus***	**CLZhao 29713**	**PQ306581**	**PQ306588**	**China**	**Present study**
*Radulomyceshydnoides*	CLZhao 21632	OR096184	OR449914	China	Dong et al. (2024b)
*Radulomyceshydnoides*	CLZhao 21668	OR096185	OR449915	China	Dong et al. (2024b)
*Radulomycesmolaris*	ARAN-Fungi 2003	—	MT232311	Spain	Olariaga et al. (2020)
*Radulomycesmolaris*	ML0499	AY463459	AY586705	Sweden	Larsson et al. (2004)
*Radulomycespaumanokensis*	IMG 5985-16	MG050100	MG050110	Spain	Wang et al. (2018)
*Radulomycesrickii*	JK 951007	—	AY586706	Sweden	Larsson et al. (2004)
*Radulomycesrickii*	G1066	—	MK278543	Hungary	Varga et al. (2019)
***Radulomycessinensis***	**CLZhao 25667** *	**PQ306578**	**PQ306585**	**China**	**Present study**
*Radulomycesyunnanensis*	CLZhao 1262	OR096191	OR449917	China	Dong et al. (2024b)
*Radulomycesyunnanensis*	CLZhao 7364	OR096192	OR449918	China	Dong et al. (2024b)
*Radulomyceszixishanensis*	CLZhao 21127	ON033887	OR449922	China	Dong et al. (2024b)
*Radulotubusresupinatus*	Cui 8383	KU535660	KU535668	China	Zhao et al. (2016)
*Radulotubusresupinatus*	Cui 8462	KU535657	KU535665	China	Zhao et al. (2016)
*Scytinostromaportentosum*	EL 11-99	AF506470	AF506470	Sweden	Larsson and Larsson (2003)
*Varariagallica*	CBS 656.81	—	AF323742	France	Wagner (2001)
*Varariaochroleuca*	CBS 465.61	—	AF323743	France	Wagner (2001)
*Vesiculomycescitrinus*	EL 53-97	AF506486	AF506486	Sweden	Larsson and Larsson (2003)

**Figure 1.**
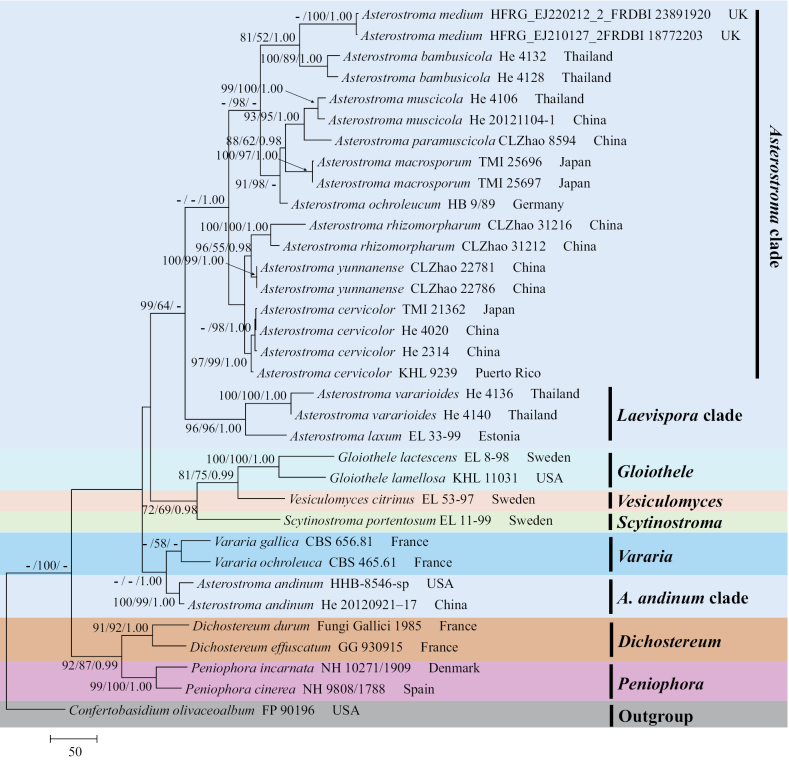
Maximum parsimony strict consensus tree illustrating the phylogeny of *Asterostroma* and related genera in the family Peniophoraceae based on ITS+nrLSU sequences. Branches are labelled with maximum likelihood bootstrap value ≥ 70%, parsimony bootstrap value ≥ 50%, and Bayesian posterior probabilities ≥ 0.95.

**Figure 2.**
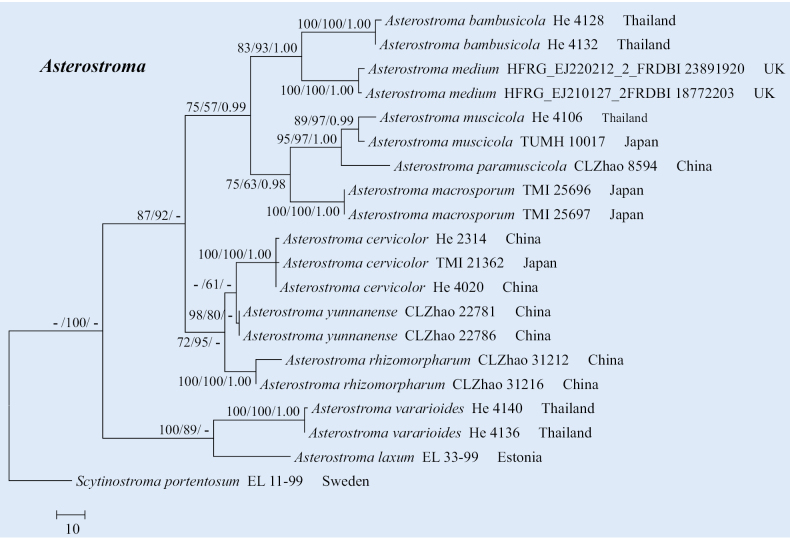
Maximum parsimony strict consensus tree illustrating the phylogeny of *Asterostromaparamuscicola* and related species in the genus *Asterostroma* based on ITS sequences. Branches are labelled with maximum likelihood bootstrap value ≥ 70%, parsimony bootstrap value ≥ 50%, and Bayesian posterior probabilities ≥ 0.95.

**Figure 3.**
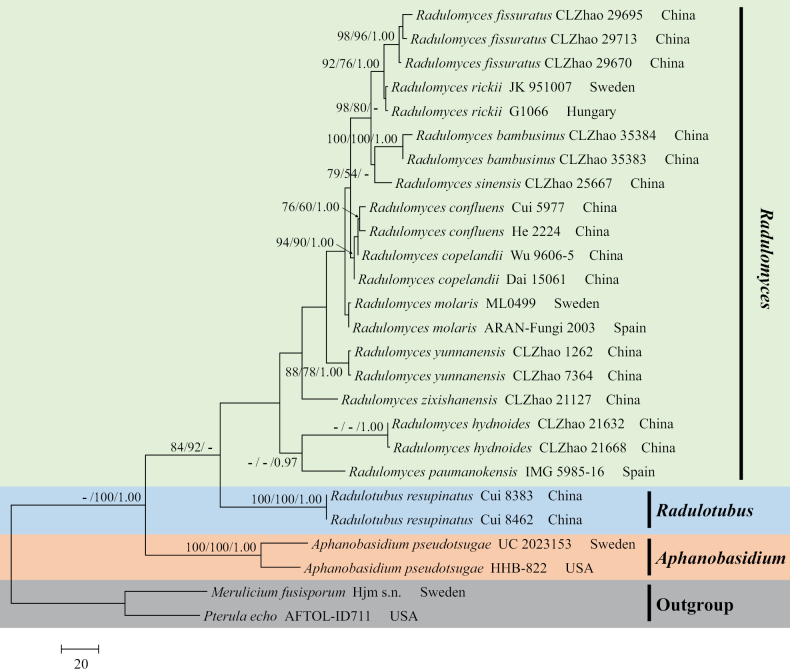
Maximum parsimony strict consensus tree illustrating the phylogeny of *Radulomyces* and related genera in the family Radulomycetaceae based on ITS+nrLSU sequences. Branches are labelled with maximum likelihood bootstrap value ≥ 70%, parsimony bootstrap value ≥ 50%, and Bayesian posterior probabilities ≥ 0.95.

**Figure 4.**
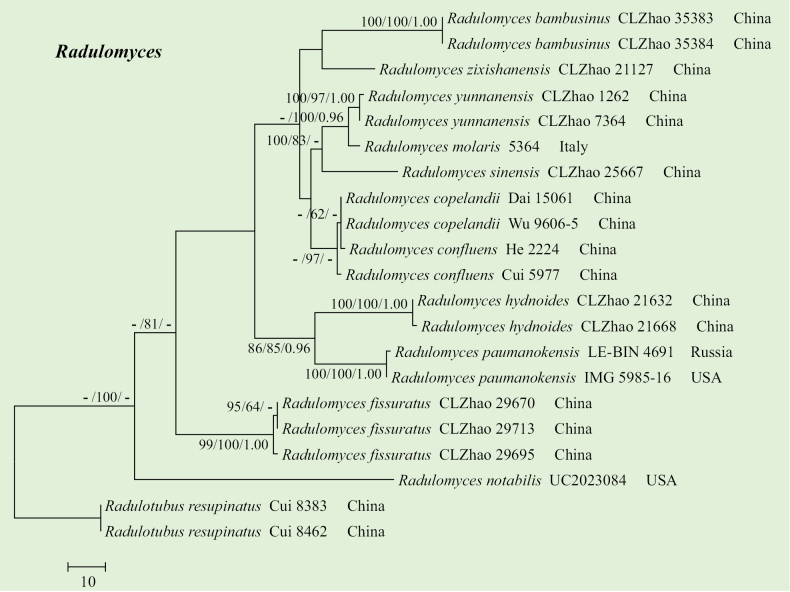
Maximum parsimony strict consensus tree illustrating the phylogeny of three new and related species in the genus *Radulomyces* based on ITS sequences. Branches are labelled with maximum likelihood bootstrap value ≥ 70%, parsimony bootstrap value ≥ 50%, and Bayesian posterior probabilities ≥ 0.95.

The sequences were aligned in MAFFT v. 7 (Katoh et al. 2019) using the G-INS-i strategy. The alignment was adjusted manually using AliView v. 1.27 (Larsson 2014). The dataset was aligned first, and then the sequences of ITS+nrLSU were combined with Mesquite v. 3.51. The combined ITS+nrLSU sequences and ITS datasets were used to infer the position of the new species and related species. The sequences of *Confertobasidiumolivaceoalbum* (Bourdot & Galzin) Jülich were retrieved from GenBank and used as outgroup taxa in the ITS+nrLSU analysis (Fig. 1) in the family Peniophoraceae; *Scytinostromaportentosum* (Berk. & M.A. Curtis) Donk was selected as the outgroup taxon for the ITS analysis (Fig. 2) in the genus *Asterostroma* (Deng et al. 2024). The sequences of *Pterulaecho* D.J. McLaughlin & E.G. McLaughlin and *Meruliciumfusisporum* (Romell) J. Erikss. & Ryvarden were selected as the outgroup taxon for the ITS+nrLSU analysis (Fig. 3) of the family Radulomycetaceae; *Radulotubusresupinatus* Y.C. Dai, S.H. He & C.L. Zhao was selected as outgroup taxa in the ITS analysis (Fig. 4) in the genus *Radulomyces* (Zhao et al. 2016).

Maximum parsimony (MP), maximum likelihood (ML), and Bayesian inference (BI) analyses were applied to the combined three datasets following a previous study (Zhao and Wu 2017), and the tree construction procedure was performed in PAUP* v. 4.0b10 (Swofford 2002). All characters were equally weighted, and gaps were treated as missing data. Trees were inferred using the heuristic search option with TBR branch swapping and 1000 random sequence additions. Max trees were set to 5000, branches of zero length were collapsed, and all parsimonious trees were saved. Clade robustness was assessed using bootstrap (BT) analysis with 1000 replicates (Felsenstein 1985). Descriptive tree statistics, tree length (TL), consistency index (CI), retention index (RI), rescaled consistency index (RC), and the homoplasy index (HI) were calculated for each maximum parsimonious tree generated. The multiple sequence alignment was also analysed using Maximum Likelihood (ML) in RAxML-HPC2 on XSEDE v. 8.2.8 with default parameters (Miller et al. 2012). Branch support (BS) for ML analysis was determined by 1,000 bootstrap replicates.

jModelTest v. 2 (Darriba et al. 2012) was used to determine the best-fit evolutionary model for each data set for Bayesian inference (BI), which was performed using MrBayes 3.2.7a (Ronquist et al. 2012). The first one-fourth of all generations was discarded as burn-in. The majority rule consensus tree of all remaining trees was calculated. Branches were considered as significantly supported if they received maximum likelihood bootstrap value (BS) ≥ 70%, maximum parsimony bootstrap value (BT) ≥ 70%, or Bayesian posterior probabilities (BPP) ≥ 0.95.

## ﻿Results

### ﻿Molecular phylogeny

The aligned dataset comprised 34 specimens representing 23 species. Four Markov chains were run for two runs from random starting trees, each for one million generations for the combined ITS+nrLSU (Fig. 1) data set with trees and parameters sampled every 1000 generations. The dataset had an aligned length of 2206 characters, of which 1453 characters are constant, 276 are variable and parsimony uninformative, and 477 are informative. Maximum parsimony analysis yielded nine equally parsimonious trees (TL = 1735, CI = 0.6317, HI = 0.3683, RI = 0.6751, and RC = 0.4265). The best model for the ITS+nrLSU dataset, estimated and applied in the Bayesian analysis, was GTR+I+G. Both Bayesian analysis and ML analysis resulted in a similar topology to MP analysis, with an average standard deviation of split frequencies = 0.004237 (BI), and the effective sample size (ESS) for Bayesian analysis across the two runs is double the average ESS (avg. ESS) = 505.

The aligned dataset comprised 20 specimens representing 11 species. Four Markov chains were run for two runs from random starting trees, each for 0.5 million generations for the ITS (Fig. 2) data set with trees and parameters sampled every 1000 generations. The dataset had an aligned length of 620 characters, of which 384 characters are constant, 51 are variable and parsimony uninformative, and 185 are informative. Maximum parsimony analysis yielded one equally parsimonious tree (TL = 412, CI = 0.7694, HI = 0.2306, RI = 0.8450, and RC = 0.6502). The best model for the ITS dataset, estimated and applied in the Bayesian analysis, was HKY+G. Both Bayesian analysis and ML analysis resulted in a similar topology to MP analysis with an average standard deviation of split frequencies = 0.004683 (BI), and the effective sample size (ESS) for Bayesian analysis across the two runs is double the average ESS (avg. ESS) = 435.

The aligned dataset comprised 26 specimens representing 15 species. Four Markov chains were run for two runs from random starting trees, each for one million generations for the combined ITS+nrLSU (Fig. 3) data set with trees and parameters sampled every 1000 generations. The dataset had an aligned length of 2115 characters, of which 1699 characters are constant, 132 are variable and parsimony uninformative, and 284 are informative. Maximum parsimony analysis yielded 143 equally parsimonious trees (TL = 711, CI = 0.7496, HI = 0.2504, RI = 0.7623, and RC = 0.5715). The best model for the ITS+nrLSU dataset, estimated and applied in the Bayesian analysis, was GTR+I+G. Both Bayesian analysis and ML analysis resulted in a similar topology to MP analysis, with an average standard deviation of split frequencies = 0.008051 (BI), and the effective sample size (ESS) for Bayesian analysis across the two runs is double the average ESS (avg. ESS) = 535.5.

The aligned dataset comprised 21 specimens representing 12 species. Four Markov chains were run for two runs from random starting trees, each for 0.5 million generations for the ITS (Fig. 4) data set with trees and parameters sampled every 1,000 generations. The dataset had an aligned length of 671 characters, of which 444 characters are constant, 74 are variable and parsimony uninformative, and 153 are informative. Maximum parsimony analysis yielded ten equally parsimonious trees (TL = 373, CI = 0.7587, HI = 0.2413, RI = 0.8109, and RC = 0.6153). The best model for the ITS dataset, estimated and applied in the Bayesian analysis, was HKY+G. Both Bayesian analysis and ML analysis resulted in a similar topology to MP analysis, with an average standard deviation of split frequencies = 0.006832 (BI), and the effective sample size (ESS) for Bayesian analysis across the two runs is double the average ESS (avg. ESS) = 346.5.

The phylogram, based on the combined ITS+nrLSU sequences (Fig. 1) analysis, showed that the new species, *Asterostromaparamuscicola*, was assigned to the genus *Asterostroma* within the family Peniophoraceae. The phylogenetic tree, based on ITS sequences (Fig. 2), revealed that *A.paramuscicola* was closely associated with *A.macrosporum* N. Maek. & Suhara. and *A.muscicola* (Berk. & M.A. Curtis) Massee. The phylogram, based on the combined ITS+nrLSU sequences (Fig. 3) analysis, showed that three new species, *Radulomycesbambusinus*, *R.fissuratus*, and *R.sinensis*, were assigned to the genus *Radulomyces* within the family Radulomycetaceae. The phylogenetic tree, based on ITS sequences (Fig. 4), revealed that *R.bambusinus* was retrieved as a sister to *R.zixishanensis*. The taxon *R.fissuratus* forms a monophyletic lineage. The other species, *R.sinensis*, was closely associated with *R.molaris* (Chaillet ex Fr.) M.P. Christ. and *R.yunnanensis* J.H. Dong & C.L. Zhao.

### ﻿Taxonomy

#### ﻿ Asterostroma paramuscicola

Taxon classificationFungiRussulalesPeniophoraceae

J.H. Dong & C.L. Zhao sp. nov.

D4CC099B-89CB-5692-A5D2-54F8E684685D

 855659

Figs 5A , 6 , 7

##### Holotype.

China • Yunnan Province, Pu’er, Jingdong County, Taizhong Town, Ailaoshan Ecological Station, GPS coordinates 24°31'N, 101°02'E, altitude 2400 m asl., a dead angiosperm tree, leg. C.L. Zhao, 24 August 2018, CLZhao 8594 (SWFC).

**Figure 5.**
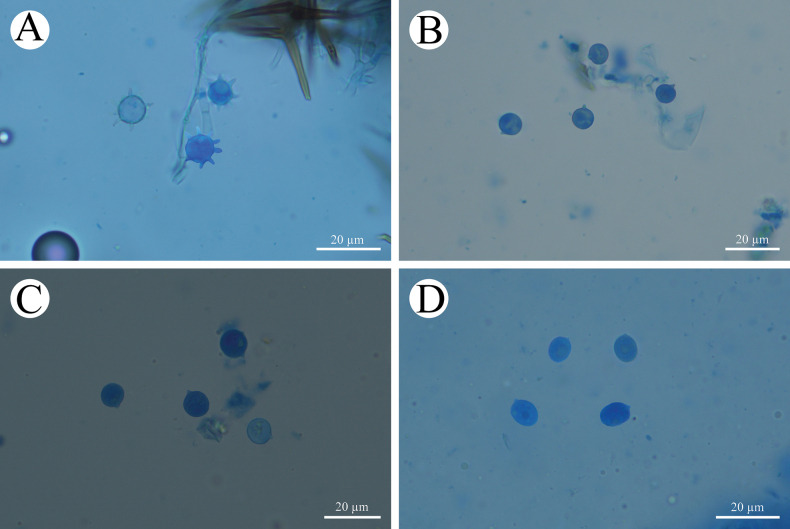
Sections of hymenium of *Asterostroma* and *Radulomyces***A***Asterostromaparamuscicola* (holotype, CLZhao 8594) **B***Radulomycesbambusinus* (holotype, CLZhao 35383) **C***Radulomycesfissuratus* (holotype, CLZhao 26970) **D***Radulomycessinensis* (holotype, CLZhao 25667). 10 × 100 Oil.

##### Etymology.

*paramuscicola* (Lat.): referring to its close phylogenetic relationship with *A.muscicola*.

##### Basidiomata.

Annual, resupinate, felted-membranous to pellicular, soft, without odour and taste when fresh, becoming coriaceous upon drying, up to 10 cm long, 5 cm wide, 50–150 µm thick. Hymenial surface smooth, pinkish when fresh, turning to pinkish to slightly salmon-buff upon drying, rhizomorphic. Sterile margin thin, cream to buff, fimbriate, up to 1 mm wide.

**Figure 6.**
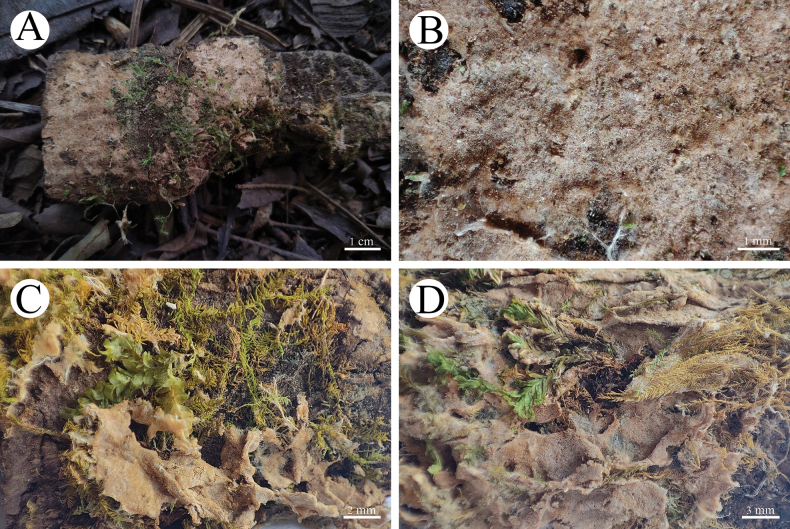
Basidiomata of *Asterostromaparamuscicola* in general and detailed views (holotype, CLZhao 8594).

##### Hyphal system.

Dimitic, generative hyphae scattered, simple-septate, colorless, thin-walled, occasionally branched, 2–3.5 µm in diameter, IKI–, CB–, tissues unchanged in KOH. Asterosetae in subiculum abundant, predominant, yellowish brown, thick-walled, regularly star-shaped, 4–6 µm in diameter, weakly dextrinoid, rays up to 75 µm long, with acute tips, CB–, and tissues unchanged in KOH.

##### Hymenium.

Asterohyphidia in hymenium similar to asterosetae in subiculum, but smaller and less regularly shaped, 2–3.5 µm in diameter, rays up to 25 µm long, usually bifurcated at tips. Gloeocystidia subcylindrical to fusiform, thin-walled, with a basal simple septum, 45–83.5 × 9–21.5 µm; cystidioles absent. Basidia subcylindrical, colorless, with four sterigmata and a basal simple septum, 47.5–58 × 7.5–9.5 µm; basidioles dominant, similar to basidia in shape, but slightly smaller.

**Figure 7.**
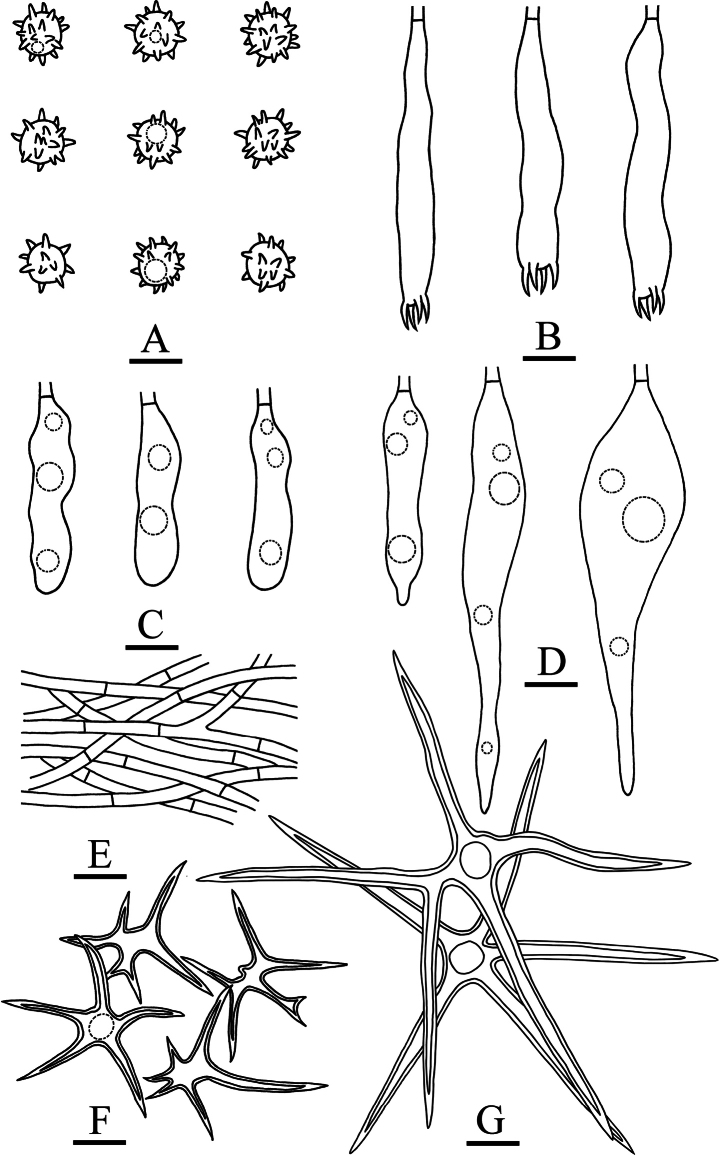
Microscopic structures of *Asterostromaparamuscicola* (holotype, CLZhao 8594) **A** basidiospores **B** basidia **C** basidioles **D** gloeocystidia **E** generative hyphae **F** asterohyphidia from hymenium **G** asterosetae from subiculum. Scale bars: 10 µm (**A–G**).

##### Basidiospores.

Subglobose, with a distinct apiculus, spines conical, 2–3 µm long, colorless, thin-walled, echinulate, amyloid, with one guttula, CB–, (7.5–)8–8.8(–9) × (6.6–)7–8(–8.5) µm, L = 8.39 µm, W = 7.65 µm, Q = 1.02–1.22, Q_m_ = 1.10 ± 0.06 (n = 30/1).

#### ﻿ Radulomyces bambusinus

Taxon classificationFungiRussulalesPeniophoraceae

J.H. Dong & C.L. Zhao sp. nov.

C0569954-6C9B-59B1-88B1-CD52CFB5663D

 855660

Figs 5B , 8 , 9

##### Holotype.

China • Yunnan Province, Zhaotong, Daguan County, Wumengshan National Nature Reserve, GPS coordinates 27°46'N, 103°52'E, altitude 2200 m asl., on the dead bamboo, leg. C.L. Zhao, 6 November 2023, CLZhao 35383 (SWFC).

**Figure 8.**
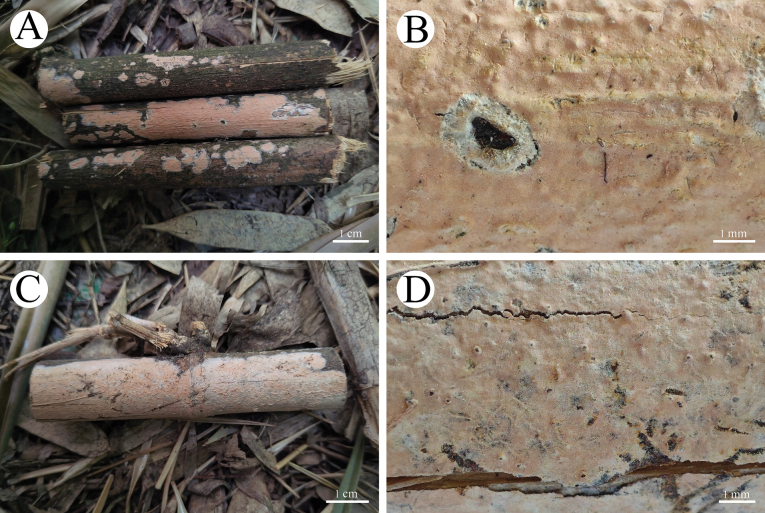
Basidiomata of *Radulomycesbambusinus* in general and detailed views **A, B** CLZhao 35383 (holotype) **C, D** CLZhao 35384.

##### Etymology.

*bambusinus* (Lat.): referring to the type species growing on bamboo.

##### Basidiomata.

Annual, resupinate, adnate, soft membranous, without odour or taste, becoming coriaceous upon drying, up to 5 cm long, 2 cm wide, 50–100 µm thick. Hymenial surface tuberculate, cream to pinkish when fresh, turning to pinkish-white to pink upon drying. Sterile margin cream to slightly pinkish, thinning out, up to 1 mm wide.

**Figure 9.**
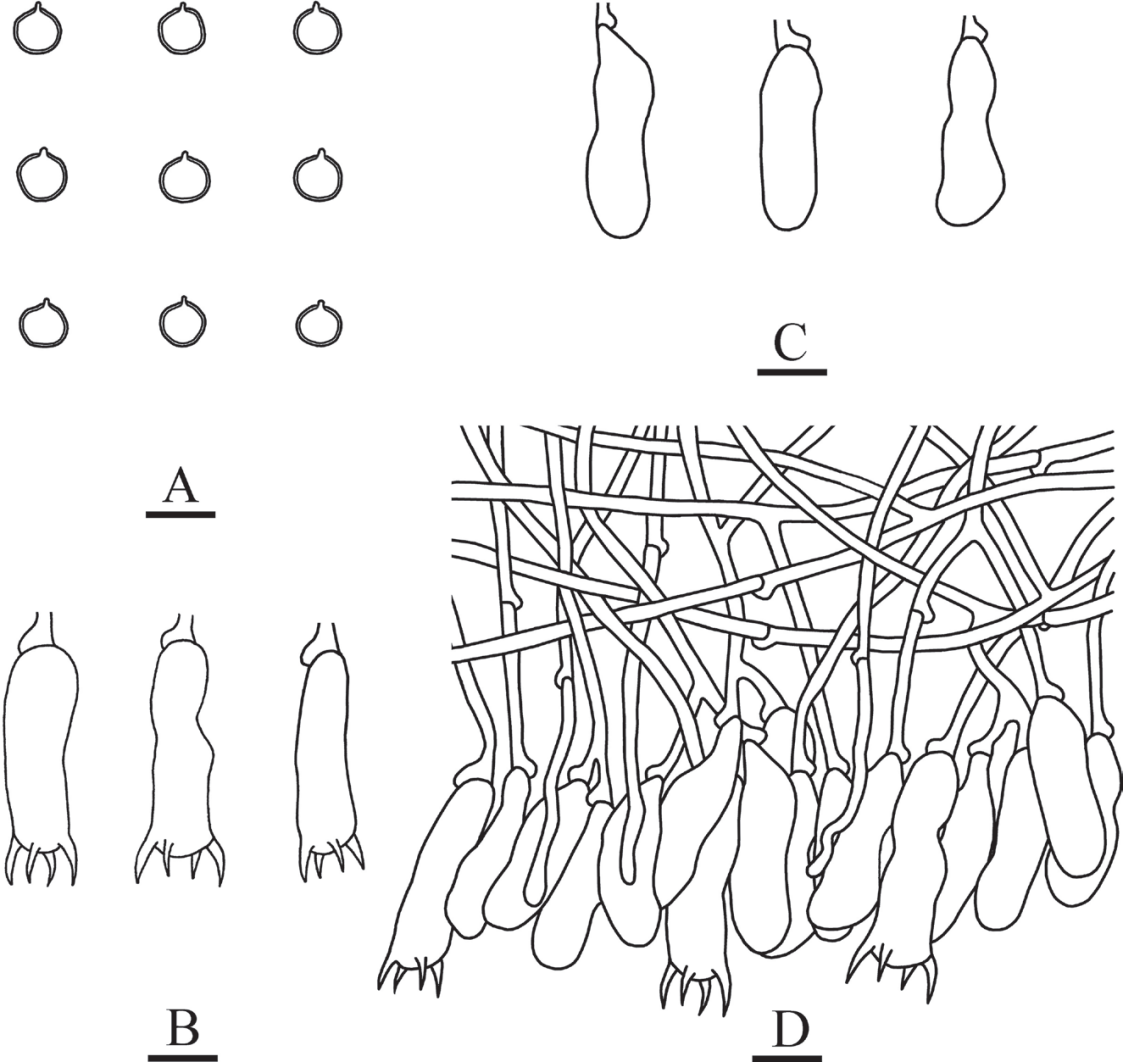
Microscopic structures of *Radulomycesbambusinus* (holotype, CLZhao 35383) **A** basidiospores **B** basidia **C** basidioles **D** part of the vertical section of hymenium. Scale bars: 10 µm (**A–D**).

##### Hyphal system.

Monomitic, generative hyphae with clamp connections, colorless, thin-walled, branched, interwoven, 2.5–3.5 µm in diameter; IKI–, CB–, tissues unchanged in KOH.

##### Hymenium.

Cystidia and cystidioles absent. Basidia clavate to barrelled, with 4 sterigmata and a basal clamp connection, occasionally constricted in the middle, 23.5–40.5 × 7.5–10.5 µm; basidioles dominant, similar to basidia in shape, but slightly smaller.

##### Basidiospores.

Subglobose, slightly thick-walled, smooth, colorless, CB+, (5.7–)6–7.5(–8) × 5.5–7.3(–7.5) µm, L = 6.76 µm, W = 6.43 µm, Q = 1.01–1.18, Q_m_ = 1.05 ± 0.05 (n = 60/2).

##### Additional specimen (paratype) examined.

China • Yunnan Province, Zhaotong, Daguan County, Wumengshan National Nature Reserve, GPS coordinates 27°46'N, 103°52'E, altitude 2200 m asl., on the dead bamboo, leg. C.L. Zhao, 6 November 2023, CLZhao 35384 (SWFC).

#### ﻿ Radulomyces fissuratus

Taxon classificationFungiRussulalesPeniophoraceae

J.H. Dong & C.L. Zhao sp. nov.

BE0A8C4E-E968-52AD-9BD2-86E461585795

 855661

Figs 5C , 10 , 11

##### Holotype.

China • Yunnan Province, Dehong, Yingjiang County, Tongbiguan Provincial Nature Reserve, GPS coordinates 23°48'N, 97°38'E, altitude 1000 m asl., on the fallen branch of angiosperm, leg. C.L. Zhao, 17 July 2023, CLZhao 29670 (SWFC).

**Figure 10.**
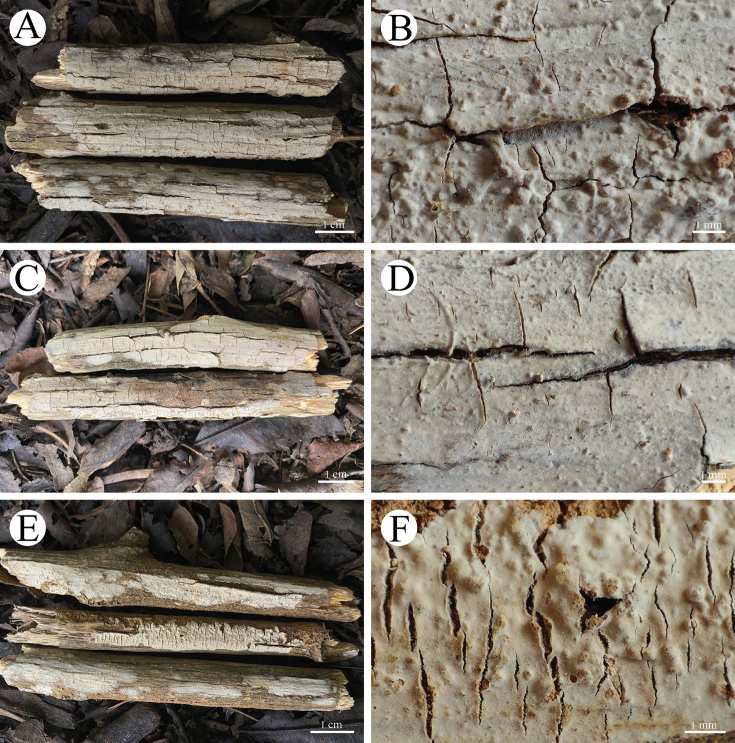
Basidiomata of *Radulomycesfissuratus* in general and detailed views **A, B**, CLZhao 29670 (holotype) **C, D** CLZhao 29713 **E, F** CLZhao 29695.

##### Etymology.

*fissuratus* (Lat.): referring to the cracked hymenial surface of the type specimen.

##### Basidiomata.

Annual, resupinate, adnate, membranaceous, without odour or taste, becoming hard coriaceous upon drying, up to 30 cm long, 2 cm wide, 50–150 µm thick. Hymenial surface tuberculate, cream when fresh, turning grey to grey-buff upon drying. Sterile margin cream, thinning out, up to 1 mm wide.

##### Hyphal system.

Monomitic, generative hyphae with clamp connections, colorless, thin-walled, branched, interwoven, 2–3.5 µm in diameter; IKI–, CB–, tissues unchanged in KOH.

**Figure 11.**
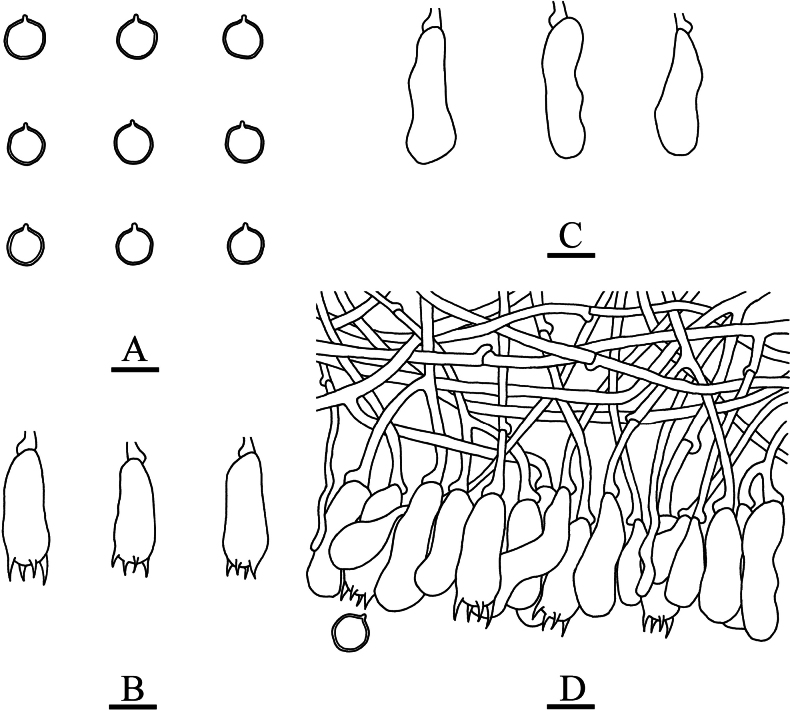
Microscopic structures of *Radulomycesfissuratus* (holotype, CLZhao 29670) **A** basidiospores **B** basidia **C** basidioles **D** part of the vertical section of hymenium. Scale bars: 10 µm (**A–D**).

##### Hymenium.

Cystidia and cystidioles absent. Basidia clavate to barrelled, with 4 sterigmata and a basal clamp connection, 21.5–32.5 × 8.5–10.5 µm; basidioles dominant, similar to basidia in shape, but slightly smaller.

##### Basidiospores.

Globose, slightly thick-walled, smooth, colorless, CB+, (6.5–)7–9(–9.5) × (6.2–)6.5–8.5(–8.8) µm, L = 8.07 µm, W = 7.76 µm, Q = 1.01–1.06, Q_m_ = 1.04 ± 0.03 (n = 90/3).

##### Additional specimens (paratypes) examined.

China • Yunnan Province, Dehong, Yingjiang County, Tongbiguan Provincial Nature Reserve, GPS coordinates 23°48'N, 97°38'E, altitude 1000 m asl., on the fallen branch of angiosperm, leg. C.L. Zhao, 17 July 2023, CLZhao 29695; CLZhao 29713 (SWFC).

#### ﻿ Radulomyces sinensis

Taxon classificationFungiRussulalesPeniophoraceae

J.H. Dong & C.L. Zhao sp. nov.

1ADEB193-683A-580C-B944-A1A553086FC5

 855662

Figs 5D , 12 , 13

##### Holotype.

China • Yunnan Province, Lincang, Fengqing County, Yaojie Town, Xingyuan Village, GPS coordinates 24°58'N, 99°92'E, altitude 1600 m asl., on the fallen branch of angiosperm, leg. C.L. Zhao, 22 October 2022, CLZhao 25667 (SWFC).

**Figure 12.**
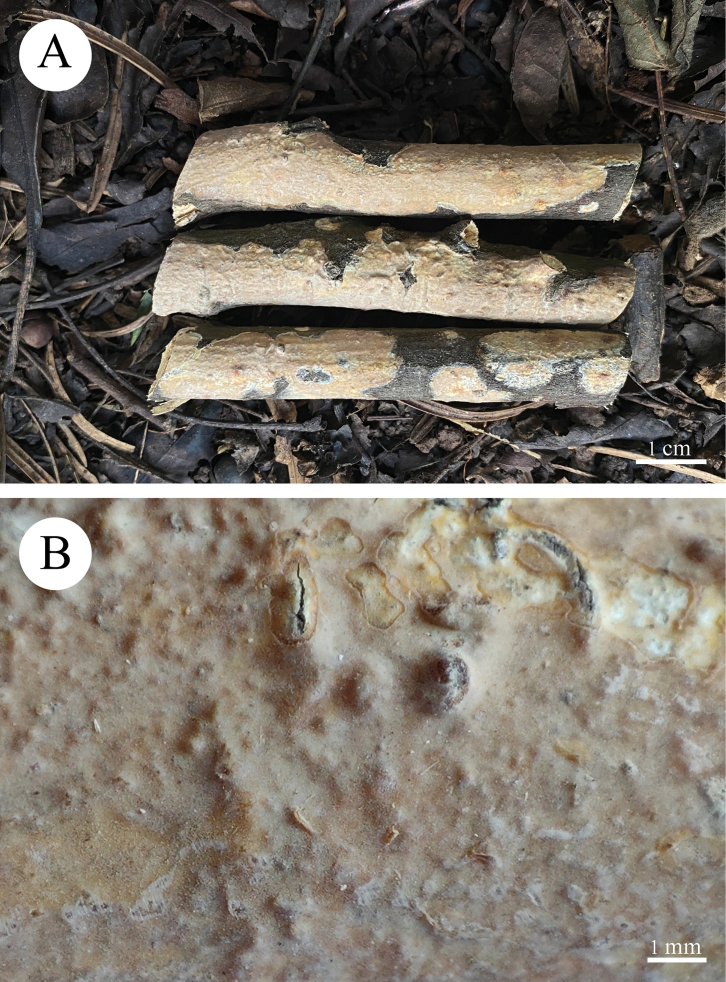
Basidiomata of *Radulomycessinensis* in general and detailed views (holotype, CLZhao 25667).

##### Etymology.

*sinensis* (Lat.): referring to the locality (China) of the type specimen.

##### Basidiomata.

Annual, resupinate, adnate, soft coriaceous, without odour or taste, becoming hard coriaceous upon drying, up to 20 cm long, 2 cm wide, 100–150 µm thick. Hymenial surface tuberculate, buff to slightly straw when fresh, turning to straw to cinnamon to ocherous upon drying. Sterile margin cream to slightly straw, abrupt, up to 1 mm wide.

**Figure 13.**
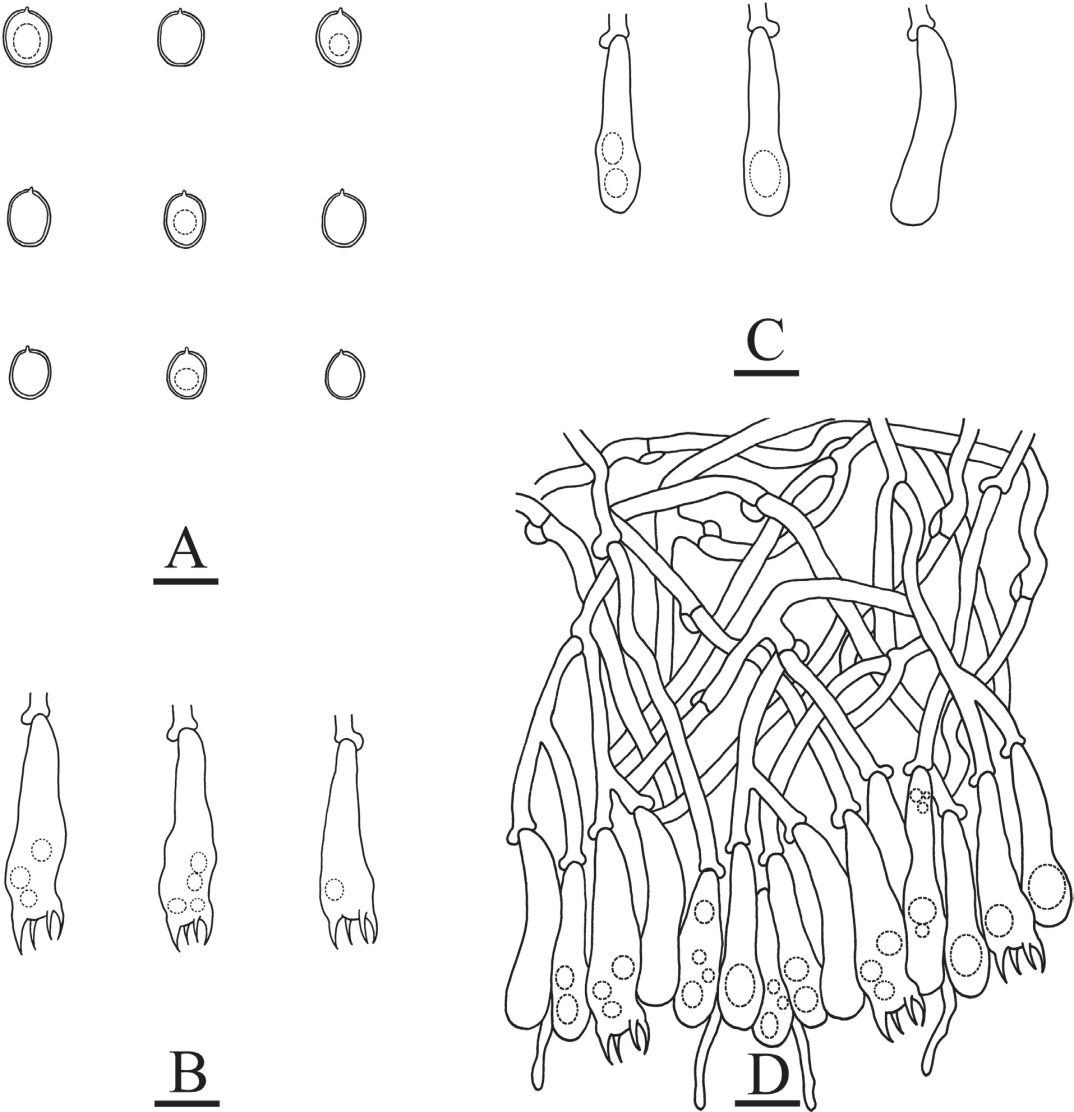
Microscopic structures of *Radulomycessinensis* (holotype, CLZhao 25667) **A** basidiospores **B** basidia **C** basidioles **D** part of the vertical section of hymenium. Scale bars: 10 µm (**A–D**).

##### Hyphal system.

Monomitic, generative hyphae with clamp connections, colorless, thin-walled, branched, interwoven, 2.5–3.5 µm in diameter; IKI–, CB–, tissues unchanged in KOH.

##### Hymenium.

Cystidia and cystidioles absent. Basidia clavate, with 4 sterigmata and a basal clamp connection, with a median constriction, 35–41.5 × 7.5–9.5 µm; basidioles dominant, similar to basidia in shape, but slightly smaller.

##### Basidiospores.

Broadly ellipsoid, slightly thick-walled, smooth, colorless, some with guttulae, CB+, 7.5–9(–9.5) × (5.8–)6.2–7.5(–7.7) µm, L = 8.42 µm, W = 6.88 µm, Q = 1.10–1.33, Q_m_ = 1.23 ± 0.08 (n = 30/1).

## ﻿Discussion

In the present study, four new species, *Asterostromaparamuscicola*, *Radulomycesbambusinus*, *R.fissuratus*, and *R.sinensis*, are described based on phylogenetic analyses and morphological characteristics.

*Asterostroma* is a monophyletic genus in our phylogenetic analysis with low statistical support, in contrast to the previous study (Liu et al. 2017; Zhou et al. 2024). Seven species with ornamented basidiospores formed the section Asterostroma as *A.bambusicola* S.L. Liu & S.H. He, *A.cervicolor* (Berk. & M.A. Curtis) Massee, *A.macrosporum*, *A.medium* Bres., *A.muscicola*, *A.ochroleucum*, *A.paramuscicola*, *A.rhizomorpharum*, and *A.yunnanense*, while three species with smooth basidiospores formed two clades as the *A.andinum* Pat. clade (only *A.andinum*) and sect. Laevispora (*A.laxum* Bres. and *A.vararioides* S.L. Liu & S.H. He).

Phylogenetically, the phylogram based on the combined ITS+nrLSU sequences (Fig. 1) analysis showed that the new species *Asterostromaparamuscicola* was assigned to the genus *Asterostroma* within the family Peniophoraceae. The phylogenetic tree, based on ITS sequences (Fig. 2), revealed that *Asterostromaparamuscicola* was closely associated with *A.macrosporum* and *A.muscicola*. However, the morphological characteristics of *A.macrosporum* differ from *Asterostromaparamuscicola* by having an ochreous to fulvous hymenial surface (Suhara et al. 2010b). The species *A.muscicola* differentiates from *A.paramuscicola* by having a salmon hymenial surface, smaller basidia (18–24 × 5–6 µm), and shorter basidiospores (6–8 × 5.5–7.5 µm; Boidin et al. 1997).

The phylogram, based on the combined ITS+nrLSU sequences (Fig. 3) analysis, showed that three new species, *Radulomycesbambusinus*, *R.fissuratus*, and *R.sinensis*, were assigned to the genus *Radulomyces* within the family Radulomycetaceae. The phylogenetic tree, based on ITS sequences (Fig. 4), revealed that *Radulomycesbambusinus* was retrieved as a sister to *R.zixishanensis*. The taxon *R.fissuratus* forms a monophyletic lineage. The other one species, *R.sinensis*, was closely associated with *R.molaris* and *R.yunnanensis*. However, the morphological characteristics of *R.zixishanensis* differ from *R.bambusinus* by having a cream to slightly brown, smooth hymenial surface and ellipsoid, thin-walled, basidiospores (7–8.8 × 5.5–6.8 µm; Dong et al. 2024b). The species *R.molaris* differs from *R.sinensis* by having a yellowish to cream, hydnoid hymenial surface (Bernicchia and Gorjón 2010). The species *R.yunnanensis* can be distinguished from *R.sinensis* by having a cream, smooth hymenial surface, shorter basidia (24–35 × 7–11 µm), and thin-walled basidiospores (8.2–9.5 × 5.5–7 µm; Dong et al. 2024b).

*Asterostromaparamuscicola*, *A.rhizomorpharum*, and *A.yunnanense* are all described from China. However, *A.rhizomorpharum* can be distinguished from *A.paramuscicola* in its cream to buff, cracked hymenial surface, shorter basidia (30–45 × 5.5–8 µm), and smaller basidiospores (5.5–6.8 × 4.6–5.9 µm; Zhou et al. 2024). The species *A.yunnanense* can be distinguished from *A.paramuscicola* in its cream to salmon-buff hymenial surface, smaller basidia (31–38 × 4–5 µm), and smaller basidiospores (4.5–6 × 4–5 µm; Deng et al. 2024).

Morphologically, *Asterostromaparamuscicola* resembles *A.bambusicola*, *A.boninense* Suhara & N. Maek., and *A.vararioides* in sharing a fimbriate basidiomata and subglobose to globose basidiospores. However, *A.bambusicola* differs from *A.paramuscicola* by having a brownish yellow, grayish brown, light brown, to brownish-red hymenial surface, shorter basidia (30–45 × 5.5–8 µm; Liu et al. 2017). The species *A.boninense* differentiates from *A.paramuscicola* by having the buff to partly ochreous hymenial surface and shorter basidiospores (5.5–7.5 × 5–7.2 µm; Suhara et al. 2010a). The species *A.vararioides* can be distinguished from *A.paramuscicola* in its grayish brown, light brown, to dark brown hymenial surface and smooth, smaller basidiospores (6–7 × 5.5–6 µm; Liu et al. 2017).

*Radulomycesbambusinus* shares similarities with *R.yunnanensis* and *R.zixishanensis* in having a cracked hymenial surface. However, *R.yunnanensis* can be distinguished from *R.bambusinus* by its smooth hymenial surface, ellipsoid, thin-walled, shorter basidiospores (8.2–9.5 × 5.5–7 µm; Dong et al. 2024b). The species *R.zixishanensis* differentiates from *R.bambusinus* by having the smooth hymenial surface, ellipsoid, thin-walled basidiospores (7–8.8 × 5.5–6.8 µm; Dong et al. 2024b).

*Radulomycesfissuratus* resembles *R.copelandii* (Pat.) Hjortstam & Spooner, *R.hydnoides*, and *R.paumanokensis* J. Horman, Nakasone & B. Ortiz in sharing subglobose to globose, slightly thick-walled basidiospores. However, *R.copelandii* differs from *R.fissuratus* due to its white hymenial surface, smaller basidia (29–35 × 6–7 µm), and basidiospores (6.4–7 × 5.4–6.2 µm; Ginns and Millman 2011). The species *R.hydnoides* can be distinguished from *R.fissuratus* by its hydnoid hymenial surface and shorter basidia (21–34 × 8.5–12.5 µm; Dong et al. 2024b). The species *R.paumanokensis* differentiates from *R.fissuratus* by having a hydnoid hymenial surface, smaller basidia (25–31 × 5–7.5 µm), and shorter basidiospores (5.8–6.9 × 5.2–6.4 µm; Wang et al. 2018).

*Radulomycessinensis* shares similarities with *R.arborifer* Malysheva & Zmitr., *R.molaris*, and *R.zixishanensis* in having ellipsoid basidiospores. However, *R.arborifer* differs from *R.sinensis* due to its dendroid or coralloid hymenial surface (Malysheva 2006). The species *R.molaris* can be distinguished from *R.sinensis* by its hydnoid hymenial surface (Bernicchia and Gorjón 2010). The species *R.zixishanensis* differentiates from *R.sinensis* by having the cream to slightly brown, smooth hymenial surface and thin-walled basidiospores (7–8.8 × 5.5–6.8 µm; Dong et al. 2024b).

In recent years, the wood-inhabiting fungi have been an extensively studied group of Basidiomycota, which includes a number of poroid, smooth, grandinoid, odontioid, and hydnoid basidiomata in China (Liu et al. 2023; Mao et al. 2023; Zhao et al. 2023; Dong et al. 2024b; Wang et al. 2024; Zhao et al. 2024). This paper enriches our knowledge of fungal diversity in China. We anticipate that more undescribed wood-inhabiting fungi taxa will be discovered throughout China after extensive collection combined with morphological and molecular analyses.

## Supplementary Material

XML Treatment for Asterostroma paramuscicola

XML Treatment for Radulomyces bambusinus

XML Treatment for Radulomyces fissuratus

XML Treatment for Radulomyces sinensis

## ﻿Additional information

### Author ORCIDs

Junhong Dong https://orcid.org/0000-0001-8740-0805

Qiaohua Deng https://orcid.org/0009-0002-4683-2702

Minglan Chen https://orcid.org/0009-0006-7890-2604

Daxiang Chen https://orcid.org/0009-0001-2309-1991

Chunqin Zhou https://orcid.org/0009-0004-8117-1537

Changlin Zhao https://orcid.org/0000-0002-8668-1075

### Data availability

All of the data that support the findings of this study are available in the main text.
